# Supplementary material for: Automated Nanodroplet Dispensing for Large-Scale Spheroid Generation via Hanging Drop and Parallelized Lossless Spheroid Harvesting
Source: Micromachines (Basel). 2024 Jan 31;15(2):231. doi: 10.3390/mi15020231 (PMC10893090; doi:10.3390/mi15020231)
Supplement: Supplementary file 1 [file micromachines-15-00231-s001.zip › micromachines-2767087-supplementary (pdf.io).pdf]

micromachines

MDPI

Article

Supplementary Materials: Automated nanodroplet dispensing for large-scale spheroid generation via hanging-drop and parallelized lossless spheroid harvesting

Viktoria Zieger [1,*], Ellen Wöhr [2,3], Stefan Zimmermann [1], Daniel Frejek [2], Peter Koltay [1], Roland Zengerle [1,2] and Sabrina Kartmann [1,2]

1 Laboratory for MEMS Applications, IMTEK- Department of Microsystems Engineering, University of Freiburg, Georges-Koehler-Allee 103, D-79110 Freiburg, Germany
2 Hahn-Schickard, Georges-Koehler-Allee 103, D-79110 Freiburg, Germany [3]
University of Furtwangen, Germany
* Correspondence: Viktoria.zieger@imtek.uni-freiburg.de

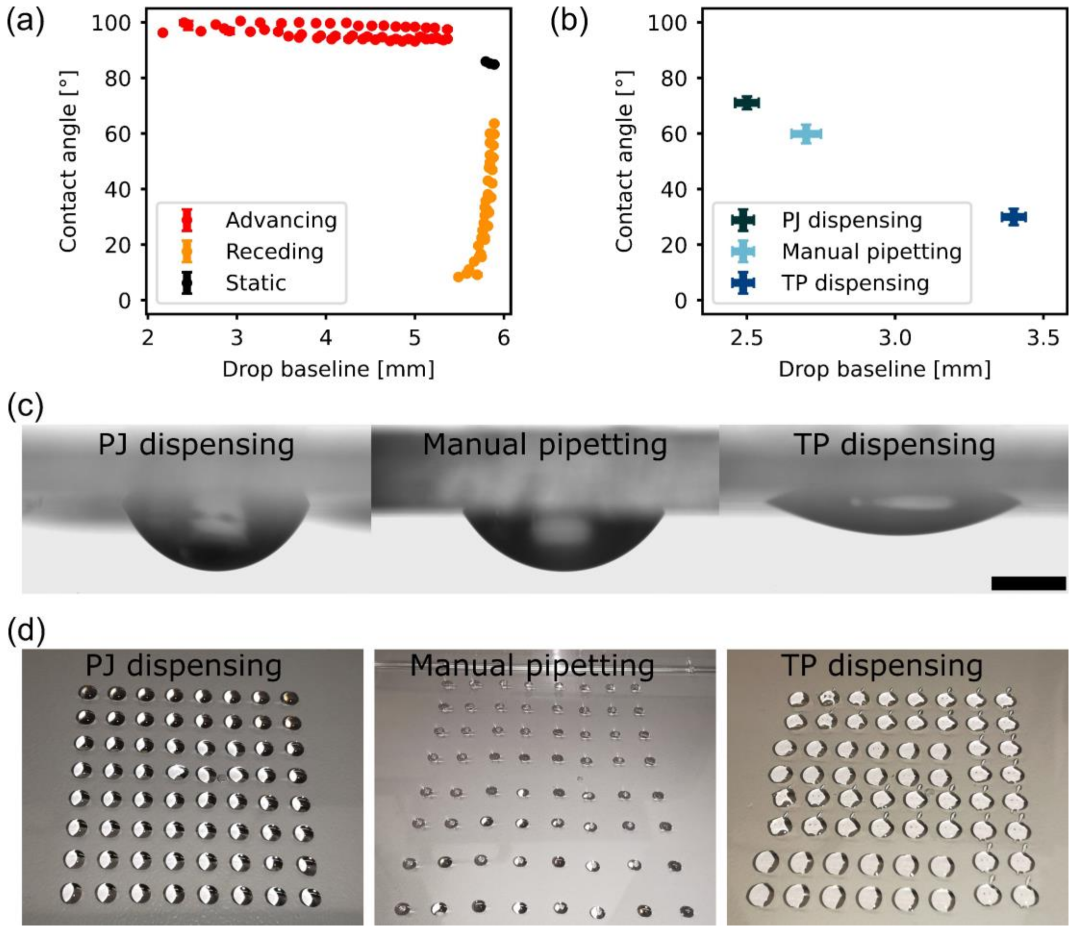

Figure S1. Geometry of complete spheroid medium drops: (a) Measurement of the advancing, receding and static contact angles between complete spheroid medium and a standard polystyrene lab dish using the needle method. (b) Measurement of the established contact angles of 2 µl drops of the complete spheroid medium depending on different dispensing methods. (c) Exemplary shapes of 2 µl complete spheroid medium depending on the applied dispensing method. Scale bar: 1 mm. (d) Representative drop arrays produced with different dispensing methods.

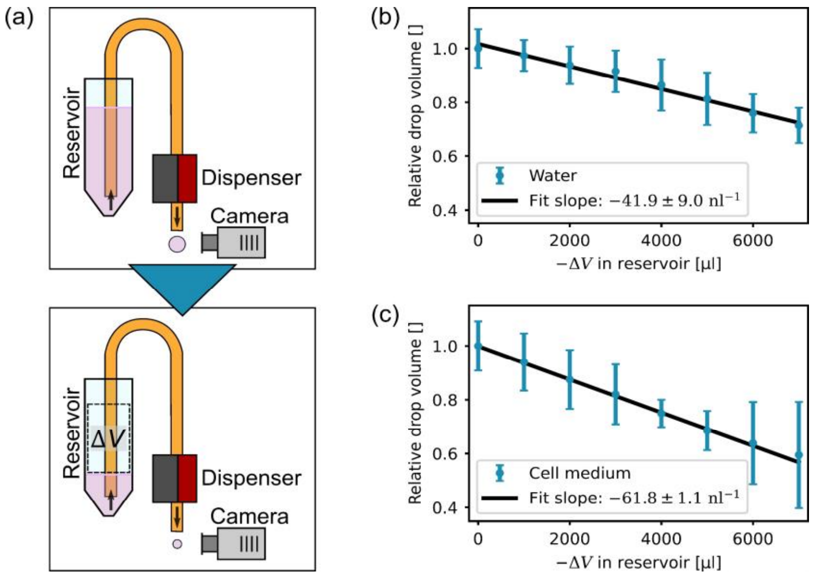

Figure S2. Dependency of dispensed nanoliter droplet volume on hydrostatic pressure: (a) Depending on the filling level in the reservoir and with that the hydrostatic pressure in the capillary, the ejected droplet varies. (b) Decrease of the ejected drop volume with decreasing reservoir volume for water. (c) Decrease of the ejected drop volume with decreasing reservoir volume for complete spheroid medium. For (b) and (c) each data point contains at least 20 different measurements.

Supplementary text: Automated volume correction for decreasing reservoir volume

As the level of cell suspension in the reservoir decreases, the droplet volume of each droplet dispensed also decreases due to the reduced hydrostatic pressure (Figure S2a). To maintain a constant droplet volume, one could use a pressure-regulated reservoir. However, this would hinder and complicate the mixing and single-use nature of all components in contact with the sample. Therefore, we measured the relationship between the fill level in the reservoir and the resulting droplet volume. As expected, a linear relationship was observed (Figure S2b). Interestingly, the slope of the linear curve depends on the type of liquid (Figure S2b,c), which is associated with different surface tension, viscosity and wettability effects that play a role in droplet detachment from the nozzle. For large scale spheroid generation, typically a few milliliters of cell suspension are processed and the reduced dispensed volume would have measurable effects on spheroid sizes. Online measurement of the dispensed droplet volume would slow down the printing process and require a greater distance between the target substrate and the nozzle orifice. The latter may have a negative effect on the droplet shape.

In order to account for the reduced volume per drop dispensed and to dispense the same volume for each hanging drop on the substrate, we use the measured slope from Figure S2c as a correction factor. With the measured slope, we can calculate and automatically adjust the number n of drops required to produce a hanging drop of the desired volume during a long print run:

$$n = \frac{V_H}{V_d} = \frac{V_H}{V_i - f \cdot x \cdot V_H \cdot V_i}, \tag{1}$$

where V_H is the target volume of the hanging drop, f is the slope of the curve, V_i is the initial drop volume measured for a full reservoir, x is the number of already printed hanging drops and V_d is the actual droplet volume.

In general, Equation 6 results in more droplets with a smaller volume being dispensed for the last hanging drop generated than for the first hanging drop in a non-stop printing process

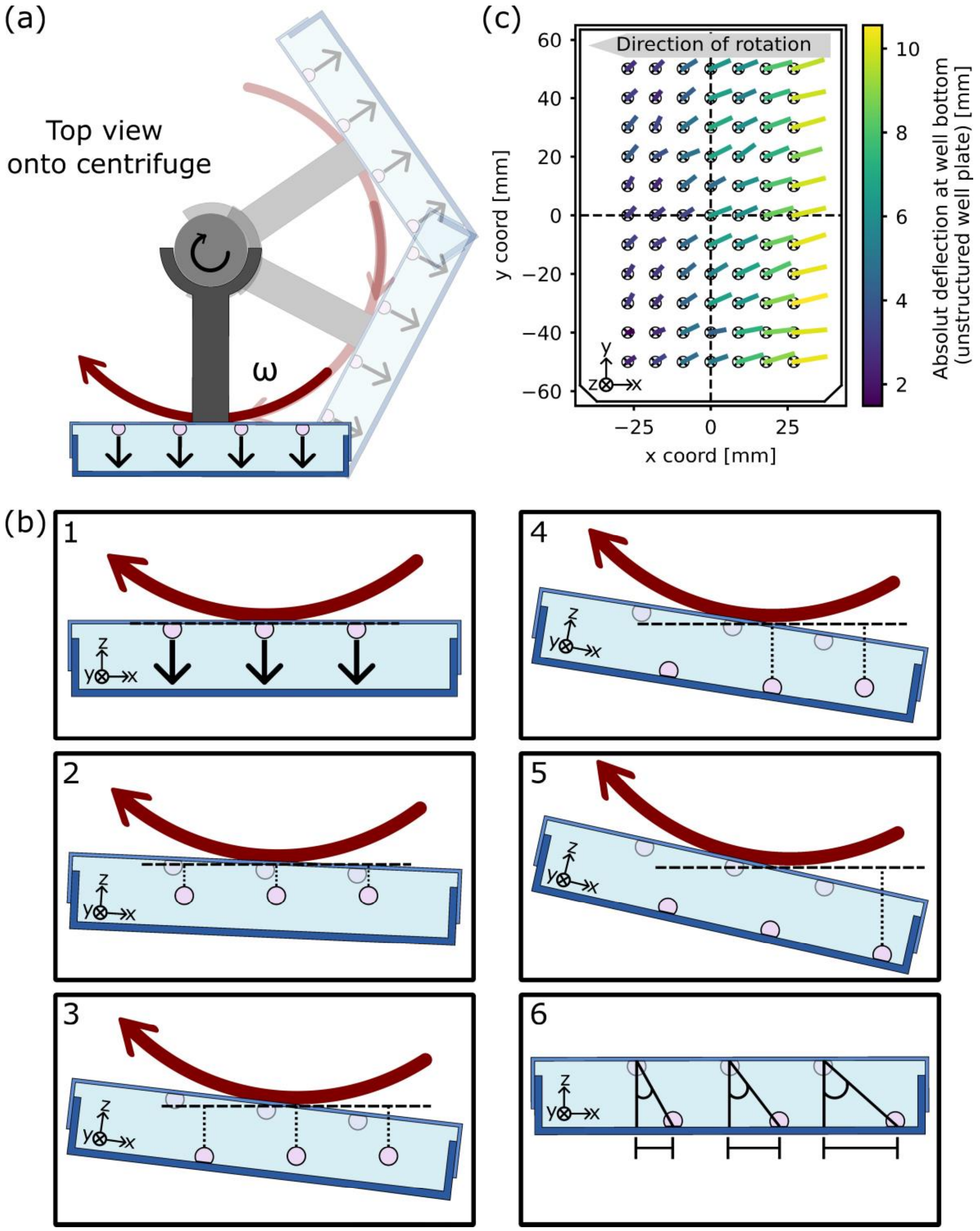

Figure S3. Drop deflection during centrifugation: (a) Schematic top view on a centrifuge rotator with an unstructured microplate with hanging drops in the lid. The arrows indicate the direction of the centrifugal force vector, which always points radially outwards. (b) Schematic representation of the trajectories of detached drops in a centrifuge. As the donor and receiver plates continue to rotate and the drops follow a radially outward directed trajectory, the displacement of the impact position on the receiver plate depends on the initial position of the hanging drop on the donor plate with respect to the direction of rotation. (c) Measured deflection of 2 µl drops from a donor lid into an unstructured one-well plate at 25 g. The arrows indicate the direction and magnitude of the deflection. Because the centrifugal acceleration is very low, the plate carrier does not swing completely to a vertical position, resulting in a slight deflection of the drop also in y.
